# Effects of Pazopanib Monotherapy vs. Pazopanib and Topotecan Combination on Anaplastic Thyroid Cancer Cells

**DOI:** 10.3389/fonc.2019.01202

**Published:** 2019-11-12

**Authors:** Teresa Di Desidero, Paola Orlandi, Daniela Gentile, Guido Bocci

**Affiliations:** Dipartimento di Medicina Clinica e Sperimentale, University of Pisa, Pisa, Italy

**Keywords:** pazopanib, topotecan, anaplastic thyroid cancer, synergism, gene expression

## Abstract

The purpose of this study was to examine pazopanib/topotecan combination activity vs. pazopanib monotherapy on anaplastic thyroid cancer (ATC) cells. Proliferation analyses were performed on ATC cell lines administered for 72 h with pazopanib and topotecan alone and to their simultaneous combination. Pazopanib and topotecan produced a strong synergism on ATC cells, calculated by the combination index, increasing the intracellular concentrations of topotecan lactone measured by high-performance liquid chromatography. Furthermore, a significantly decrease of the gene expression of *ATP-binding cassette transporter G2 (ABCG-2), vascular endothelial growth factor (VEGF), hypoxia-inducible factor-1*α *(HIF-1*α*)*, and *colony stimulating factor-1 (CSF-1)* was presented in combination-treated ATC cells by real time PCR tests. In summary, the simultaneous association of pazopanib and topotecan established a highly synergistic ATC antiproliferative effect, suggesting a new possibility to translate this schedule into clinical trials.

## Introduction

Anaplastic thyroid cancer (ATC) is a rare, but aggressive form of thyroid tumor with very short survival and poor prognosis (1). ATC is always refractory to conventional therapies including surgery, standard radiotherapy and chemotherapy (2). In fact, these therapeutic strategies are not advantageous in improving the survival rates of patients affected by thyroid cancer (1, 3). Thus, identifying alternative treatment options is a crucial point for ATC management. Current research suggests that chemotherapeutic drugs have poor or absence of activity toward ATC if given alone (1, 3). In the past decade, the discovery of genetic and molecular characteristics fundamental for the evolution of thyroid cancers drove to the progress of new treatment agents, in particular the tyrosine kinase inhibitors (TKIs). Some of them, such as vandetanib, cabozantinib, sorafenib, and lenvatinib obtained effective results in phase III studies on medullary thyroid carcinoma and differentiated thyroid carcinoma, but it is still in debate if they could have a role in the treatment of ATC patients (3). Although some preclinical experiments have shown an *in vitro* and *in vivo* antitumor effect in ATC by TKIs (4–7), their monotherapy revealed a poor efficacy, as demonstrated by pazopanib or imatinib (8, 9). Certainly, Bible et al. described an absence of RECIST responses in pazopanib-treated ATC patients, even though a transitory disease regression was seen (10). To our knowledge, no preclinical findings have been published on the combo treatment of pazopanib and topotecan, a camptothecin derivative, in ATC. There are many inspiring reasons to perform this preclinical study on ATC. New combination therapeutic approaches are urgently needed for this rare but aggressive neoplasm. Thus, efforts in this sense should be supported such as the choice of novel combinations never tested before but with a solid rationale. In this perspective, our group have investigated the *in vitro* and *in vivo* activity in ATC of the camptothecin analog irinotecan (11), a drug belonging to the same therapeutic class of topotecan. Moreover, the combination of irinotecan and sunitinib, a TKI like pazopanib, was highly synergistic, suggesting a possible successful association between camptothecins and angiokinase inhibitors that was never tested before in ATC. Furthermore, our group previously collaborated to investigate the combination of topotecan plus pazopanib in other preclinical models such as primary or late stage metastatic triple-negative breast cancer and in metastatic renal cell carcinoma, where we found that this combination was particularly effective and promising (12, 13). Based on these considerations, it was natural to suggest the translation of topotecan/pazopanib also into the ATC field.

The aims of the present study were (1) to examine whether the combined schedule of pazopanib and topotecan were synergistic and (2) to establish the possible underlying mechanism of this effect looking at the intracellular concentration of the drugs.

## Materials and Methods

### Materials and Drugs

The cell culture media RPMI, supplements and all other chemicals were purchased from Sigma Aldrich SRL (Milan, Italy). Quantitative real-time PCR reagents were acquired from Applied Biosystems (Foster City, CA, USA). Topotecan and pazopanib were bought from Selleckchem (DBA Italia, Milan, Italy), and prepared in a stock solution of 10 mM in 100% dimethylsulfoxide (DMSO) for cell's experiments. Control's medium wells were treated with the same DMSO concentration used to formulate the medium of the wells with the highest concentration of pazopanib and topotecan in the same experiment.

### Cell Lines

The human ATC cell line 8305C (14) (BRAF V600E mutated) was purchased from DSMZ (Braunschweig, Germany, DSMZ no.: ACC 133), instead the human ATC cell line FB3 (6) (HRAS Q61R mutated) was a gift from Prof. Fulvio Basolo (University of Pisa). Both cell lines were grown in RPMI 1640 medium supplemented with 15% FBS and L-glutamine (2 mM). The cells were used for experiments at the fourth passage.

### Antiproliferative Assay

The antiproliferative assay is a standard method to detect the inhibition of the cultured cancer cell proliferation. In particular, the assay has been performed to test the pharmacological effects of topotecan or pazopanib on 8305C and FB3 cell line growth because they have never been described before in the scientific literature.

*In vitro* antiproliferative effect was verified on 8305C and FB3 cell lines, as previously described (6, 15). Briefly, 8305C and FB3 cells were exposed for 72 h (1 × 10^4^ cells/well) to pazopanib (0.1–100 μM) or topotecan (0.01–1,000 nM) or with their vehicle as control. The half maximal inhibitory concentration (IC_50_) was calculated by a non-linear regression fit of the mean values.

### *In vitro* Assessment of Synergistic Effect of Pazopanib and Topotecan on ATC Cells

The *in vitro* assessment of synergism between pazopanib and topotecan on ATC cells has been performed with the objective (i) to evaluate the type of pharmacological interaction between the used drugs and (ii) to suggest a possible dose reduction of each drug used in the combination schedules (16).

The association of topotecan (0.01–1,000 nM) with pazopanib (0.1–100 μM) was explored on 8305C and FB3 cells in a fixed molar concentration ratio (1:10), respectively. Briefly, the combination index (CI) method (16) was followed to calculate the level of interaction between topotecan and pazopanib, where CI < 1, CI = 1, and CI > 1 indicate synergism, additive effect, and antagonism, respectively. The CI has been calculated as follows:

CI = [(D)_1_/(D_X_)_1_] + [(D)_2_/(D_X_)_2_]

To exemplify the process, at the 75% suppression level, (Dx)_1_ and (Dx)_2_ are the concentrations of pazopanib and topotecan, respectively, that induce a 75% inhibition of cell proliferation; instead (D)_1_ and (D)_2_ are the concentrations of pazopanib and topotecan in association that also block cell proliferation by 75% (isoeffective as compared with the single drugs alone).

The dose reduction index (DRI) represents the theoretical magnitude of concentration reduction permitted for each drug when administered in synergistic combination to obtain the same result as that acquired with the concentration of each single agent:

(DRI)_1_ = (D_X_)_1_/(D)_1_ and (DRI)_2_ = (D_X_)_2_/(D)_2_

The CI and DRI indexes were calculated using CalcuSyn v.2.0 software (Biosoft, Cambridge, UK).

### Intracellular Accumulation of Topotecan in ATC Cells

The high performance liquid chromatography (HPLC) is one of the most common techniques used for the determination of drug content in different matrices such as plasma, urine, or cell lysates, over different periods of time. In our study, the HPLC analysis has the fundamental aim to investigate the intracellular accumulation of topotecan lactone after the exposure to single and combined drugs, providing quantitative results.

The quantitative analysis of topotecan lactone, the active metabolite of topotecan, in ATC cells (8305C and FB3) was executed as previously described (12, 17). Briefly, 8305C and FB3 cells (1 × 10^6^) were incubated with topotecan (1 μM) in 15% FBS RPMI in 10 cm^2^ dishes at 37°C. After incubation, the treatment medium was discharged and cells were washed with cold PBS (pH 5), scraped and ultrasonically lysed (10 s × 3). The cell lysate (200 μL) was mixed to 400 μL cold methanol and centrifuged (7,500 g) for 5 min at 4°C to extract topotecan lactone. Proteins of the lysate were quantified using the Bio-Rad colorimetric analysis (Bio-Rad Laboratories). The sample concentration of topotecan was determined by a previously described sensitive HPLC method (12, 17). Topotecan was diluted in pH 3 PBS at 37°C to convert it in the active lactone as calibration standard.

### Modulation of *ABCG2, VEGF, HIF-1α*, and *CSF-1* Gene Expression

The real-time polymerase chain reaction (PCR) is a widely used, highly accurate and sensitive technique for investigating gene expression levels (18). To evaluate the expression of the human *ABCG2, VEGF, HIF-1*α, and *CSF-1* genes, 8305C and FB3 cells were treated with topotecan and pazopanib or in simultaneous association at a concentration corresponding to their experimental IC_50_ or with vehicle alone for 72 h. We have chosen these genes since their important role in the ATC cancer cells and their changes could be involved in the synergistic effects of the simultaneous combination of pazopanib and topotecan in ATC cells, as reported in the discussion section of the text. Quantitative RT-PCR was performed with the Applied Biosystems 7900HT sequence detection system (Applied Biosystems, Carlsbad, CA, USA) as previously described (19). Briefly, RNA (1 μg) was reverse transcribed and the resulting complementary DNA was diluted (2:3) and amplified. *ABCG2, VEGF, HIF-1*α and *CSF1* validated primers were purchased from Applied Biosystems (*ABCG2*, Assay ID Hs01053796_m1, *VEGF* Hs00170236_m1, *HIF-1*α HS00936368_m1, and *CSF-1* Hs00174164_m1). Manufacturer's instructions were adopted for PCR thermal cycling settings and optimization of primer concentrations. Amplifications were standardized to glyceraldehyde 3-phosphate dehydrogenase (GAPDH), and the measurement of gene expression was achieved using the ΔΔCt calculation, where Ct was the threshold cycle; the quantity of target, standardized to the endogenous control and relative to the calibrator (vehicle-treated control cells), was given as 2^−ΔΔCt^. All experiments were independently performed three times with at least nine samples for each concentration.

### Statistical Analysis

Results (mean ± SEM) from the performed experiments were subjected to statistical analysis and in particular to the analysis of variance between groups (ANOVA), followed by the Student–Newman–Keuls test. The level of significance was set at *P* < 0.05. GraphPad Prism software package version 5.0 (GraphPad Software, Inc., San Diego, CA) was used to perform the above-mentioned analysis.

## Results

### Pazopanib and Topotecan Inhibit ATC Cells Proliferation *in vitro*

**Figure 1:**
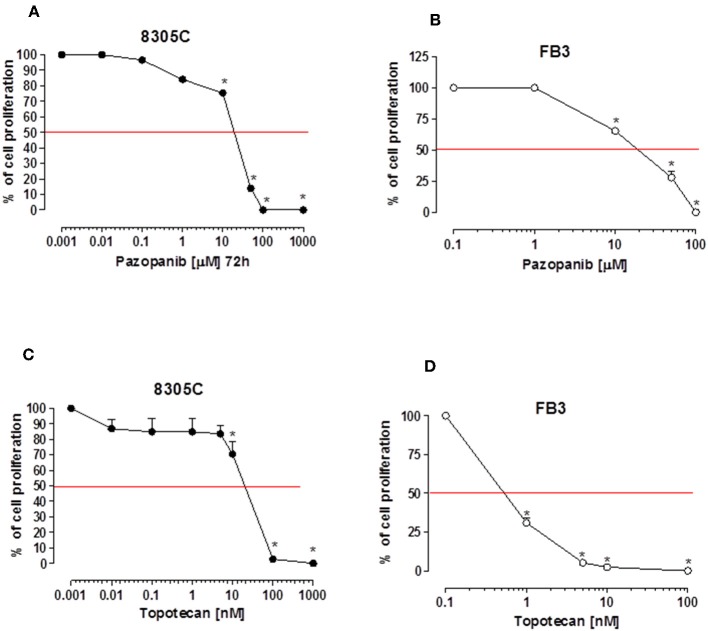
Antiproliferative effects of pazopanib and topotecan *in vitro* on 8305C (**A**, **C**, respectively) and FB3 cell lines (**B**, **D**, respectively). The antiproliferative effect of the drug was studied after 72 h of exposure. The data are presented as percentage of vehicle-treated cells. The data were obtained in triplicate experiments (i.e., at least 9 wells for each concentration). Symbols and bars, mean values ± SEM, respectively. ^*^P < 0.001 vs. control.

Pazopanib suppressed *in vitro* cell proliferation of 8305C and FB3 cell lines in a concentration-dependent manner. The exposure to pazopanib for 72 h arrested the 8305C and FB3 cell proliferation with a calculated IC_50_ of 25,080 ± 3,220 nM (Figure 1A) and 18,020 ± 1,307 nM (Figure 1B), respectively.

A stronger antiproliferative effect was found using topotecan on 8305C and FB3 cell lines, as demonstrated by the calculated IC_50s_ of 307.3 ± 4.82 nM (Figure 1C) and 0.65 ± 33 nM (Figure 1D), respectively.

### Effects of Pazopanib and Topotecan Combination

**Figure 2:**
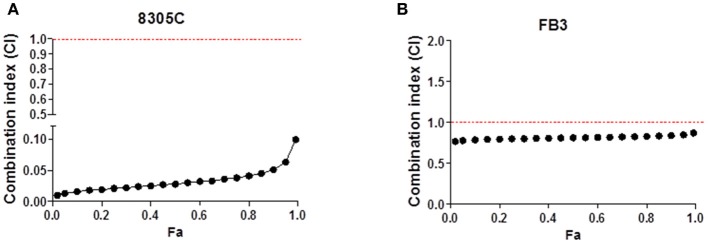
Combination index (CI)-fraction affected (Fa) plot of 72 h pazopanib and topotecan simultaneous combination in 8305C **(A)** and FB3 **(B)** cells. CI<1, CI = 1 and CI > 1 indicate synergism, additive effect, and antagonism, respectively.

A 72 h simultaneous treatment of 8305C cells to diverse combined concentrations of pazopanib and topotecan caused a very strong synergism for all the fraction of affected cells with a value of CI < 0.1 (Figure 2A). This synergism also produced a favorable DRI > 1 (Table 1). A similar effect was also obtained in FB3 cells (Figure 2B), although in a lesser extent. Indeed, the CI values ranged between 0.76 and 0.87, whereas the DRI values were favorable for topotecan but not for pazopanib (Table 1).

**Table 1:** Dose reduction index (DRI) of 72 h pazopanib and topotecan combination in 8305C and FB3 cells at 25, 50, and 75% of affected cell fraction.

	**DRI Values**					
	**25%**		**50%**		**75%**	
	**Topotecan**	**Pazopanib**	**Topotecan**	**Pazopanib**	**Topotecan**	**Pazopanib**
**FB3**	2.224	0.870	2.210	0.852	2.197	0.835
**8305C**	169.5	66.3	127	48.9	95.1	36.1

### Pazopanib Increases Intracellular Topotecan Lactone Accumulation

**Figure 3:**
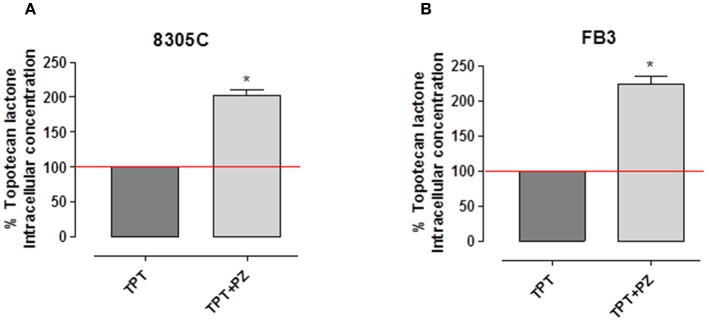
Intracellular concentration of topotecan lactone in 8305C **(A)** and FB3 **(B)** cells after treatment with topotecan (TPT) alone and with the combination of TPT and pazopanib (PZ). The data were obtained in triplicate experiments (i.e., at least 9 wells for each concentration). Columns and bars indicate the mean percentage values ± SEM vs. treated cells with topotecan alone.

Our results showed that when ATC cells were exposed to topotecan, the intracellular accumulation of topotecan lactone in both cell lines was significantly higher, ~+100%, in those treated with the association of pazopanib (Figure 3). Indeed, intracellular topotecan lactone accumulation in 8305C cells was 202.35 ± 15.32% (Figure 3A), whereas in FB3 cells was 224 ± 20.54% (Figure 3B) vs. 100% of controls (*P* < 0.0001).

### Pazopanib and Topotecan Combination Decreased *ABCG2* Gene Expression in ATC Cells

**Figure 4:**
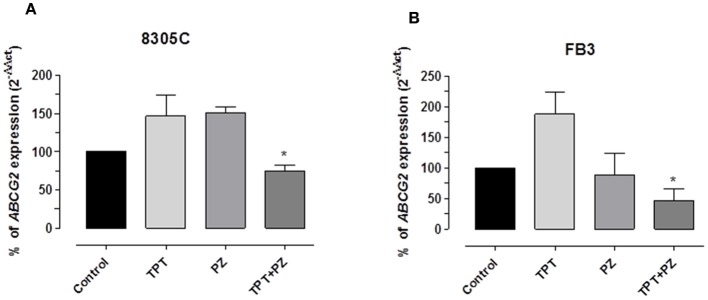
*ABCG2* gene expression in 8305C **(A)** and FB3 **(B)** cells exposed to pazopanib (PZ) and topotecan (TPT) alone or in combination, at their IC_50s_, or with vehicle alone (control) for 72 h. Data are expressed as percentage of 2^−ΔΔCt^. The data were obtained in triplicate experiments (i.e., at least 9 wells for each concentration). Columns and bars, mean values ± SEM, respectively. ^*^P < 0.05 vs. vehicle-treated controls.

Based on the synergistic effects and the intracellular accumulation of topotecan, the modulation of *ABCG2* gene expression was investigated to explore a possible relationship of this transporter and the found pharmacologic effect; the gene expression was measured in both the cell lines treated with topotecan, pazopanib and their mix at the experimental IC_50_. Figures 4A,B demonstrate a significant decline of *ABCG2* gene expression in 8305C and FB3 cells, respectively, due to the concomitant association of pazopanib and topotecan. *ABCG2* gene expression in combination-treated 8305C cells was 75.1 ± 6.94%, whereas in FB3 cells was 47.2 ± 18.4 % vs. 100% of control expression (*P* < 0.05).

### Pazopanib and Topotecan Combination Downregulate *VEGF, HIF-1α*, and *CSF-1* Gene Expression

**Figure 5:**
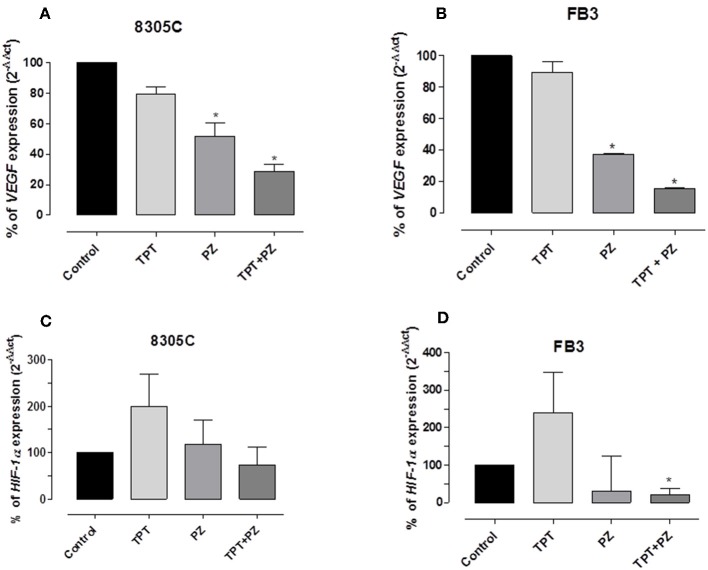
*VEGF* and HIF-1α gene expression in 8305C (**A** and **C**, respectively), and FB3 (**B** and **D**, respectively), cells exposed to pazopanib (PZ) and topotecan (TPT) alone or in combination at their IC_50s_ or with vehicle alone (control) for 72 h. Data are expressed as percentage of 2^−ΔΔCt^. The data were obtained in triplicate experiments (i.e., at least 9 wells for each concentration). Columns and bars, mean values ± SEM, respectively. ^*^P < 0.05 vs. vehicle-treated controls.

To investigate the results of pazopanib and topotecan concomitant treatment on *VEGF* and *HIF-1*α genes in 8305C and FB3 cell lines, mRNA concentrations of both genes were measured. A significant decline of *VEGF* gene expression in 8305C (Figure 5A) and FB3 (Figure 5B) cells, respectively, was found. *VEGF* gene expression in combination-treated 8305C cells was 28.72 ± 4.97%, whereas in FB3 cells was 15.7 ± 0.63% vs. 100% of control expression (*P* < 0.05).

In addition, the drug association caused reduction of *HIF-1*α expression, although not significant, in 8305 (Figure 5C), whereas this decrease reached a statistically significant difference in FB3 cells (Figure 5D). Indeed, *HIF-1*α gene expression in 8305C cell was 73.5 ± 38.36%, while in FB3 cells was 20.5 ± 17.92% vs. 100% of control expression (*P* < 0.05).

**Figure 6:**
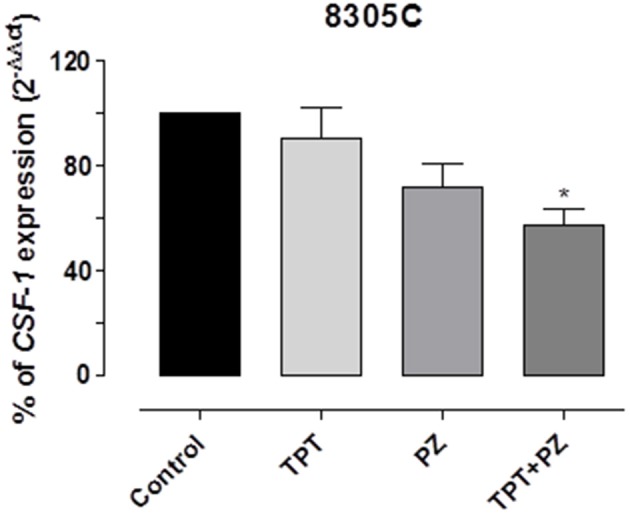
*CSF-1* gene expression in 8305C cells exposed to pazopanib (PZ) and topotecan (TPT) alone or in combination at their IC_50s_ or with vehicle alone (control) for 72 h. Data are expressed as percentage of 2^−ΔΔCt^. The data were obtained in triplicate experiments (i.e., at least 9 wells for each concentration). Columns and bars, mean values ± SEM, respectively. ^*^P < 0.05 vs. vehicle-treated controls.

Moreover, the simultaneous drug mix caused a significant decline of *CSF-1* gene expression in 8305C cells (Figure 6), when compared to the single drugs alone. Indeed, *CSF-1* gene expression in 8305C cells was 57.1 ± 10.88% vs. 100% of control expression (*P* < 0.05).

## Discussion

ATC is a rare, but extremely aggressive, undifferentiated tumor and it is responsible for the preponderance number of deaths due to thyroid cancers (20). At moment, considering that ATC is resistant to systemic radio-chemotherapy approaches (1, 21), the identification of novel therapeutic approaches is urgently needed for the ATC management of patients. This is particularly important because both phase II and retrospective clinical trials did not find any really active compound or schedule, due to the insufficient numbers of available patients (22). Even though chemotherapy can be an option for advanced/metastatic ATC patients, chemotherapeutic drugs alone revealed to be poorly efficacious (1, 23). In fact, ATC have shown minimal responses to standard chemotherapeutic drugs such as paclitaxel, mitoxantrone, cisplatin, and doxorubicin (22, 24), a topoisomerase II inhibitor, that is still in use for the therapy of the metastatic disease. A precedent of an efficacious preclinical drug association in a murine ATC model was described by the association of CPT-11 plus sunitinib (11). In a recent multi-institutional phase II trial has been described a encouraging clinical activity of the antiangiogenic TKI pazopanib in differentiated thyroid cancers patients, displaying a Response Evaluation Criteria In Solid Tumors (RECIST) response rate of 49% (25). However, in advanced ATC, although several pazopanib-treated ATC patients incurred transient disease regression, it has been described the absence of RECIST responses, suggesting a minimal single-agent clinical activity (10). Indeed, pazopanib was usually more effective in combination with standard chemotherapy in preclinical studies of different tumor types (12, 26). In this perspective, Isham et al. found that the combination of pazopanib with paclitaxel caused an elevated and synergistic activity in ATC cells *in vitro* and *in vivo* (27). Among the available chemotherapeutic drugs, the camptothecin derivative topotecan, a DNA topoisomerase I inhibitor, has revealed an interesting activity in combination with TKIs. Topotecan binds DNA complex preventing DNA replication and RNA synthesis, inducing the block of cancer cell proliferation, and it is currently administered in ovarian and non-small cell lung cancer (28). In this study, our group explored the combination of pazopanib with topotecan, and investigated the possible mechanisms involved in the antitumor effects of this therapeutic mix. Our experiments established, for the first time, that the concomitant association of pazopanib and topotecan was strongly synergistic on ATC continuous cell lines and that the combination could allow a theoretical marked reduction of doses of both drugs (as indicated by DRIs), consistently reducing their toxicity profiles. A possible limit of our study may consist in the only use of ATC continuous cell lines instead of ATC primary cell cultures. Indeed, recent papers highlighted the importance of testing ATC primary cancer cells established from biopsies (29) or tissues obtained from surgery (30) in order to evaluate the sensitivity to different TKIs, such as lenvatinib or sorafenib, in cancer cells derived from each patient, and thus to predict the response to the therapy before starting the treatment. Presently, due to a lack of data about the *in vitro* antiproliferative effect of topotecan plus pazopanib on ATC cells, a comparison could be done with a similar activity on triple negative breast cancer (12). Synergistic effects were attributable to the significant raise of intracellular concentrations of topotecan lactone, mediated by pazopanib, in ATC cells that highly augment the antiproliferative outcomes of the concomitant treatment with the two compounds. In this view, our team already proposed that the association of angiokinase inhibitors such as pazopanib, axitinib, or sunitinib with the active metabolite of irinotecan or topotecan, increase the intracellular concentrations of these camptothecins through the *ABCG2* transporter downregulation (12, 31). Several other TKIs are modulators of ABC transporters and, when combined with transporter-substrate chemotherapy agents, overcome tumor drug resistance or increase drug bioavailability (32). Moreover, ABC transporter gene overexpression plays an important role in the failure of chemotherapy by decreasing the intracellular concentration of antineoplastic drugs (33). Recently, it has been shown that the combination of antineoplastic drugs with specific TKIs, such as bafetinib and icotinib, surmounts drug resistance in various cancer cell lines (34). Therefore, the present results reinforce these prior data and propose that the concomitant association of pazopanib with topotecan increases the levels of its active metabolite inside ATC cells. Interestingly, experiments on *ABCG2* transporter have shown three hypoxia-response elements in the *ABCG2* promoter, indicating that its expression is probably controlled by *HIF*-*1*α. Indeed, those findings suggested the modulation of *ABCG2* expression by hypoxia (35), enhancing stem cell survival during treatments (36). Considering that *HIF-1*α activation may modulate *ABCG2* upregulation and that the concentration of *HIF-1*α expression may vary in different tumors, we decided to assess if pazopanib and topotecan combination could also regulate the *HIF-1*α expression decline. In fact, the data from our tests displayed also the diminished concentrations of *HIF*-*1*α mRNA and of *VEGF*, another gene target of this hypoxic factor, by the therapeutic mix of pazopanib and topotecan in both ATC cells.

In the first place, we have decided to evaluate the *ABCG2* gene since this transporter strongly affects the intracellular concentration of substrate drugs (12, 37). Our interest in *ABCG2* expression was related to the drug efflux function of this transporter that has been described as one of the mechanisms that confers multiple drug resistance to solid tumors and contribute to topotecan resistance (38). Indeed, a higher expression of this efflux pump reduces the intracellular concentrations of topotecan, declining its efficacy against its target topoisomerase I. Moreover, our previous article by Di Desidero et al. established that the combination of topotecan plus pazopanib caused a decrease of *ABCG2* expression in breast cancer cells with an increase of intracellular levels of topotecan lactone, the active form of topotecan (12). Our present findings strengthen these previous experimental data and suggest that the simultaneous combination of topotecan with pazopanib modulates the gene expression and the bioavailability of topotecan, increasing its intracellular concentration in ATC cells.

Considering that *ABCG2* upregulation is influenced by *HIF-1*α activation (39) and that the level of *HIF-1*α expression is frequently overexpressed in tumors, such as anaplastic thyroid cancer, with a strong association to the aggressive disease phenotype and the therapeutic resistance (40), we have decided to evaluate also the effects of topotecan and pazopanib combination on *HIF-1*α expression. Indeed, Melillo and his team have previously described that camptothecins, and in particular topotecan, are able to inhibit *HIF-1*α expression in preclinical models and in patients with advanced solid tumors (41). The results of our experiments showed decreased levels of mRNA HIF-1α when treated with topotecan and pazopanib in both 8305C and FB3 ATC cells.

Another target gene of *HIF-1*α is the *VEGF* one. *VEGF* expression is modulated by *HIF-1*α (42) and it is a key pro-angiogenic step for tumor progression. Moreover, pazopanib is able to block the VEGF signaling through the tyrosin kinase inhibition of VEGFR-2 (43). Thus, our interest was focused on this possible additional mechanism of action, looking also at the decrease of the receptor ligand after the simultaneous combination of two drugs.

Finally, Di Desidero et al. previously showed that the combination of the camptothecin analog irinotecan and of the TKI sunitinib caused an inhibition of *CSF-1* and *VEGF* expression in ATC cell lines (11). Indeed, CSF-1 has previously shown to induce VEGF production (44) and, as such we decided to evaluate *CSF-1* gene modulation in both 8305C and FB3 ATC cells treated with topotecan and pazopanib. In our study, we found a significant decrease in *CSF-1* gene expression levels, particularly in the simultaneous schedule of pazopanib and topotecan in ATC cells, confirming that the decline of *CSF-1* gene levels is associated with the decrease of *VEGF*.

Thyroid cancers are deeply permeated with macrophages and ATC cases displayed high tumor associated macrophages (TAMs) infiltration, which is linked to a lower survival (45). Lately, Weinberger and collaborators have identified numerous impaired molecular pathways in ATC (20). These authors founded a number of genes upregulated in ATC samples and related with TAMs, lymphocytic infiltration and macrophage phagocytosis, such as the *CSF1R* one. Indeed, ATC macrophages rely upon CSF-1 for differentiation, survival, and gene expression profile (20). Several clinical trials of CSF-1/CSF-1R inhibitors have been performed or are still open in different tumor types. TKIs, such as imatinib (46), dasatinib (47), and sunitinib (48), have been suggested to control the tumorigenic and immunosuppressive activities of TAMs. In the present study, we retrieved a significant decline of *CSF-1* gene expression after the concomitant administration of topotecan and pazopanib in our ATC cell line, suggesting that the decrease of *CSF-1* gene mRNAs could diminish the macrophages stimulation induced by this growth factor.

In conclusion, in our investigation we describe the synergistic activity of the concomitant association of pazopanib and topotecan in ATC cells. The downregulation of *ABCG2*, due to the decreased *HIF-1*α, may help to understand the found intracellular accumulation of topotecan after the administration of the combined treatment. These results may propose a possible adaptation of this therapeutic combination into future clinical trials because of both drugs are already registered and in use for other tumor types.

## Data Availability Statement

The datasets generated for this study are available on request to the corresponding author.

## Author Contributions

TD and GB designed the study. TD, PO, and DG performed the experiments and analyzed and interpreted the data. TD, DG, and GB wrote the manuscript.

### Conflict of Interest

The authors declare that the research was conducted in the absence of any commercial or financial relationships that could be construed as a potential conflict of interest. The reviewer SMF declared a past co-authorship with several of the authors TD, PO, GB to the handling editor.
